# Accelerated idioventricular rhythm observed under total intravenous anesthesia using remifentanil, propofol, and rocuronium

**DOI:** 10.1186/s40981-015-0016-3

**Published:** 2015-09-07

**Authors:** Mika Nakanishi, Kaoru Masumo, Takako Oota, Takeshi Kato, Toshihiro Imanishi

**Affiliations:** Department of Anesthesia, Osakafu Saiseikai Noe Hospital, Fruichi 1-3-25, Joto-ku, Osaka City, Osaka Japan

**Keywords:** Accelerated idioventricular rhythm, Total intravenous anesthesia, Remifentanil, Propofol

## Abstract

Accelerated idioventricular rhythm (AIVR) during anesthesia has been described in several drug toxicity such as from cocaine, halothane, desflurane, and propofol. We present the case of a man who developed episodes of AIVR observed under total intravenous anesthesia (TIVA) using remifentanil, propofol, and rocuronium. AIVR during anesthesia was a benign phenomenon, and further examinations after surgery showed no structural heart disease and the daily occurrence of idioventricular arrhythmias. This case suggests that the suppression of sinus and atrioventricular nodal function and the autonomic imbalance caused by propofol and remifentanil may induce AIVR with greater frequency.

## Background

Accelerated idioventricular rhythm (AIVR) is a ventricular rhythm comprising three or more consecutive monomorphic beats, with a gradual onset. The discharge rate of the ectopic focus is similar to the sinus rate and between 50 and 120 bpm (isorhythmic). The ectopic focus manifests when the sinus rate slows down (below that of the ectopic focus) or when the ectopic focus accelerates above its intrinsic rate by 30–40 bpm [1]. Nine clinical features are helpful in distinguishing AIVR from ventricular tachycardia (VT): chance discovery; no symptoms; no hemodynamic effects; <10 % of sinus isochronicity; HR < 120 bpm; simple conversion to sinus rhythm; short bursts; no effective drug treatment; and left bundle branch block [2].

AIVR during anesthesia has been described in several drug toxic cases such as from cocaine [3], halothane [4], desflurane [5], and propofol [6]. It might be caused by an electrolyte imbalance, post-resuscitation status after acute myocardial infarction, cardiomyopathy, or arrhythmogenic right ventricular dysplasia (ARVD) during the perioperative period [1]. We report a case of AIVR observed under total intravenous anesthesia using remifentanil, propofol, and rocuronium.

## Case presentation

**Fig. 1:**
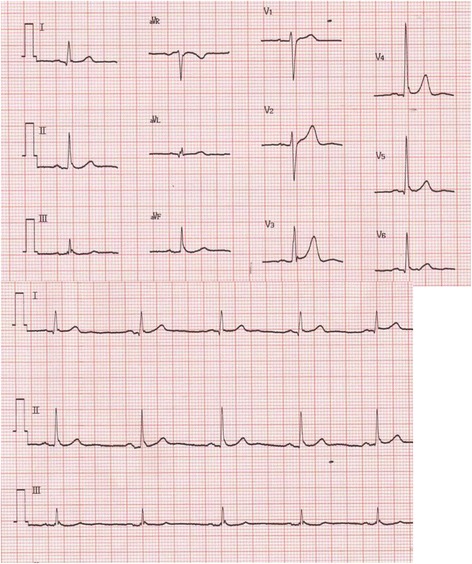
Preoperative record of a 12-lead electrocardiogram, showing a sinus rhythm of 57 bpm

A 50-year-old man, weighing 74 kg, was scheduled for arthroscopic meniscectomy for a right meniscus injury. He had undergone the same surgery on the other side 1 year prior. The previous general anesthesia was induced with sevoflurane 4 %, propofol 140 mg, fentanil 50 μg, lidocaine 50 mg, and rocuronium 50 mg. Propofol 2–3 mg/kg/h, remifentanil 0.1–0.18 μg/kg/min, and sevoflurane 1 % were maintained. His heart rate (HR) was 40–55 bpm. Atropine 0.5 mg administered intravenously did not increase his HR over 55 bpm. Idioventricular rhythm or anesthetic complication was not observed. In this time a preoperative examination revealed no sign or symptom of cardiovascular disease and he was classified as an American Society of Anesthesiologists’ (ASA) Physical Status Classification 1. A preoperative electrocardiogram (ECG) showed a sinus rhythm of 57 bpm, a QRS duration of 0.11 s, and no ventricular ectopic beat (Fig. 1).

**Fig. 2:**
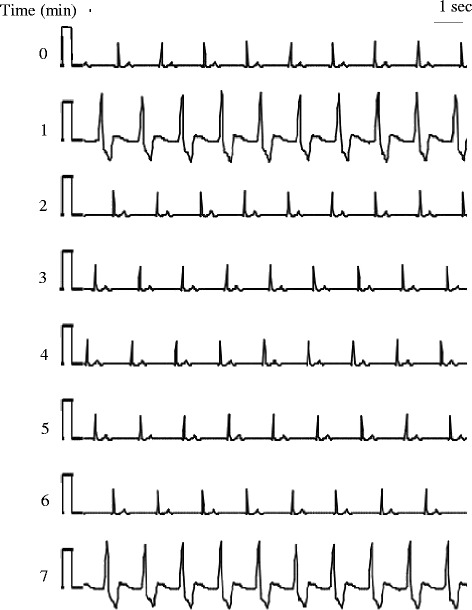
Intraoperative electrocardiogram of III lead approximately 90 min after the anesthesia start under TIVA, documenting AIVRs alternating with sinus rhythms. Ventricular arrhythmia repeated approximately every 5 min and AIVRs continued for approximately 30 s

Before anesthetic induction without premedication, the patient’s arterial blood pressure (BP) was 142/83 mmHg and his HR was 48 bpm. General anesthesia was induced with propofol 180 mg, remifentanil 0.1 μg/kg/min, and rocuronium 30 mg. After laryngeal mask insertion, atropine 0.5 mg was administered because his HR fell to 38 bpm with a sinus rhythm. Nevertheless, despite the administration of atropine 0.5 mg twice, his HR did not increase over 55 bpm. Propofol 4–5 mg/kg/h, remifentanil 0.1–0.2 μg/kg/min, and rocuronium 7 μg/kg/min were continued. His systolic BP was 90–140 mmHg, HR was 35–55 bpm, end-tidal CO_2_ was 35–40 mmHg, and SpO_2_ was 99–100 %. About 30 min after induction, ventricular arrhythmia occurred with wide QRS complexes indicative of AIVR (ventricular rhythm at this time was 48 bpm) alternating with a sinus rhythm. Ventricular arrhythmia repeated approximately every 5 min for approximately 1 h and AIVRs continued for approximately 30 s (Fig. 2). Heart rates of the idioventricular rhythms (45–50 bpm) were similar to those of the sinus rhythms at those times. The arrhythmias were independent of electric cautery and surgical procedure. Propofol, remifentanil, and rocuronium were regulated with the rate of administration mentioned above in proportion to the surgical stress. Although direct arterial sphygmomanometry in his left radial artery showed that his systolic BP was 20–30 mmHg lower during AIVRs than during sinus rhythms, his systolic BP was kept 100–120 mmHg during ventricular arrhythmia. Blood gas analyses showed plasma sodium, potassium, calcium, glucose, CO_2_, O_2_, and pH values within normal ranges. Although temporary transcutaneous pacing pads combined with a defibrillator were prepared, they were not utilized. His stable hemodynamics and short bursts of arrhythmia did not need them. The anesthetic duration was 2 h 32 min. The last AIVR just appeared when the surgery and administration of the anesthetics were finished. AIVR did not reappear after recovery from anesthesia.

During the following 24-h period, continuous ECG monitoring showed no ventricular rhythm, but sinus bradycardia at 35–50 bpm. He confessed that arrhythmia had been noted in periodical health examinations of the school and the company with no subjective symptoms and he had refused detailed examinations. Echocardiography performed 2 days later showed a normal cardiac structure. A Holter ECG 5 days after surgery showed sinus rhythms from 36–97 bpm and total rates of 0.69 % premature atrial contraction (PAC) and 6.35 % premature ventricular contraction (PVC), including an idioventricular rhythm running for 40 beats continuously. He did not have any subjective symptoms during the Holter ECG recording. The results suggested that his AIVR might have occurred daily. He refused further examinations.

## Discussion

Atrioventricular (AV) junctional rhythm, another consecutive escaped rhythm, usually has a normal QRS duration without a P wave or with a retrograde P wave. If complicated with a bundle branch block, the QRS duration gets longer. AV junctional rhythm might lack effective atrial contraction and reduce the cardiac output than sinus rhythm, because atrial contraction contributes to approximately 15 to 25 % of the diastolic filling of the ventricle [7]. AIVR originates from the His, the Purkinje system or the working contractile ventricular cells [1]. Ventricular pacing has been shown to result in dyssynchronous left ventricular (LV) electrical activation and mechanical contraction, to worsen LV ejection fraction [8]. AIVR is not artificial ventricular pacing, but its abnormal impulse conducting pathway might cause dyssynchronous LV contraction.

Some anesthetics have been described to be associated with AIVR because of toxicity. Cocaine might induce AIVR either through the production of myocardial ischemia or as a direct result of ion channel alterations [3, 9]. Halothane was reported to be associated with AIVR due to a depressant effect on slow inward calcium current and intracellular calcium accumulation [4]. Desflurane was also reported to induce AIVR because of a sympathetic imbalance [5]. Propofol was publicized to be associated with arrhythmias in humans [10]. In the guinea pig heart, the ionic mechanism underlying the negative chronotropic action of propofol on sinoatrial node (SN) automaticity was associated with propofol-induced bradycardia observed in clinical settings [11]. Similar to propofol, remifentanil also depresses sinus node function and most parameters of atrioventricular (AV) nodal function, causing remifentanil-related severe bradyarrhythmias [12]. In this case, the negative chronotropic action of propofol and remifentanil on sinus and AV nodal function might have induced the ectopic focus more often than usual.

AIVR is also associated with higher vagal tone and lower sympathetic activity [13], and the AIVR related to sympathetic nerve block during spinal anesthesia for cesarean section was reported [14]. Propofol suppresses both sympathetic and parasympathetic tone, but the suppression of sympathetic tone is more than that of parasympathetic tone [15]. Remifentanil also induces higher vagal tone [16]. In this case, superior vagal tone related to propofol and remifentanil might have induced AIVR. However, ventricular rhythm was not detected during the postoperative 24-h period including his sleeping hours, time of the parasympathetic nerve predominance. We did not succeed in clarifying the trigger of the idioventricular rhythm observed in the Holter ECG, but his activity at the time of the record was higher than that at the postoperative 24-h period. Not parasympathetic nerve predominance during sleep but imbalance of the autonomic nerve caused by anesthetics might have induced AIVR more frequently.

Naranjo algorithm is a questionnaire designed by Naranjo et al. for determining the likelihood of whether an adverse drug reaction is actually due to the drug rather than the result of other factors [17]. Probability is assigned via a score termed definite (9–13 points), probable (5–8 points), possible (1–4 points) or doubtful (0 point). On using Naranjo’s scale, we obtained a scale of 5, which makes propofol and remifentanil probable causes for the event. The adverse event appeared after their administration (+2). The adverse event improved when they were discontinued (+1). The reaction was less severe when their doses were decreased (+1), because the surgery 1 year prior had no episode of an AIVR with balanced anesthesia using sevoflurane, remifentanil, and propofol, the doses of which were lower than those at this time. The arrhythmias were objectively recorded on an ECG trace (+1).

Most AIVRs are usually well tolerated and do not need specific treatment. Increasing the sinus rate is the recommended treatment for AIVR, therefore, atropine and atrial pacing rhythm can help to control it [1, 2]. Ephedrine is controversial because it can potentially increase AIVR duration [14].

Benign AIVRs with no structural heart disease and no electrolytic abnormality, such as in this case, usually require no intervention. However, sometimes it can present as a more severe arrhythmia requiring treatment, because AIVR might be induced by reperfusion after acute myocardial infarction, some cardiomyopathies, myocarditis, and in newborn infants with various congenital heart diseases [1, 2].

## Conclusions

We observed AIVR under total intravenous anesthesia using remifentanil, propofol, and rocuronium. The suppression of sinus and atrioventricular nodal function and the autonomic imbalance caused by remifentanil and propofol might have induced AIVR with greater frequency.

## Consent

The patient’s consent to publish this case report was obtained and documented.
